# CMR-derived TAPSE measurement: a semi-quantitative method of right ventricular function assessment in patients with hypertrophic cardiomyopathy

**DOI:** 10.1007/s12471-014-0601-5

**Published:** 2014-10-08

**Authors:** C. Doesch, C. Zompolou, F. Streitner, D. Haghi, R. Schimpf, B. Rudic, J. Kuschyk, S. O. Schoenberg, M. Borggrefe, T. Papavassiliu

**Affiliations:** 1 1st Department of Medicine Cardiology, University Medical Center Mannheim, Medical Faculty Mannheim, University of Heidelberg, Theodor-Kutzer-Ufer 1-3, 68167 Mannheim, Germany; 2 DZHK (German Centre for Cardiovascular Research) partner site Mannheim, Mannheim, Germany; 3 Institute of Clinical Radiology and Nuclear Medicine, University Medical Center, Mannheim, Germany

**Keywords:** Hypertrophic cardiomyopathy, Right ventricular function, Tricuspid annular plane systolic excursion, Cardiovascular magnetic resonance

## Abstract

**Aim:**

To compare cardiovascular magnetic resonance (CMR)-derived right ventricular fractional shortening (RVFS), tricuspid annular plane systolic excursion with a reference point within the right ventricular apex (TAPSE_in_) and with one outside the ventricle (TAPSE_out_) with the standard volumetric approach in patients with hypertrophic cardiomyopathy (HCM).

**Methods and results:**

105 patients with HCM and 20 healthy subjects underwent CMR. In patients with HCM, TAPSE_in_ (*r* = 0.31, *p* = 0.001) and RVFS (*r* = 0.35, *p* = 0.0002) revealed a significant but weak correlation with right ventricular ejection fraction (RVEF), whereas TAPSE_out_ (*r* = 0.57, *p* < 0.0001) showed a moderate correlation with RVEF. The ability to predict RVEF < 45 % in HCM patients was best for TAPSE_out_. In patients with hypertrophic obstructive cardiomyopathy (HOCM), RVEF showed a significant but weak correlation with TAPSE_out_ (*r* = 0.36, *p* = 0.02) and no correlation with TAPSE_in_ (*r* = 0.05, *p* = 0.07) and RVFS (*r* = 0.02, *p* = 0.2). In patients with hypertrophic non-obstructive cardiomyopathy (HNCM), there was a moderate correlation between RVEF and TAPSE_out_ (*r* = 0.57, *p* < 0.0001) and a weak correlation with TAPSE_in_ (*r* = 0.39, *p* = 0.001) and RVFS (*r* = 0.38, *p* = 0.002). In the 20 healthy controls, there was a strong correlation between RVEF and all semi-quantitative measurements.

**Conclusion:**

CMR-derived TAPSE_in_ is not suitable to determine right ventricular function in HCM patients. TAPSE_out_ showed a good correlation with RVEF in HNCM patients but only a weak correlation in HOCM patients. TAPSE_out_ might be used for screening but the detection of subtle changes in RV function requires the 3D volumetric approach.

## Introduction

Hypertrophic cardiomyopathy (HCM) presents with a wide range of phenotypes. Apart from perturbed contractility, HCM mutations may affect energy homeostasis [1]. Besides, it can also present with right ventricular (RV) involvement. Previous studies have described an increased RV wall thickness as well as an increased RV muscle mass in a substantial proportion of patients with HCM [2–5]. Echocardiographic tissue Doppler studies also found an impairment of RV function in patients with HCM [6, 7]. Cardiovascular magnetic resonance imaging (CMR) is an image modality that combines the advantages of an excellent spatial resolution with the ability to visualise both ventricles using freely selectable image planes and has therefore emerged as gold standard for the RV volume and function measurements [8–10]. However, RV volumetry is time-consuming and requires dedicated post-processing software. In routine clinical practice, echocardiography is used to get semi-quantitative information about RV function measuring tricuspid annular systolic excursion (TAPSE). Several recent studies [11–14] using a modified TAPSE adapted from echocardiography have shown good correlation with CMR-derived RV function in a number of clinical conditions. The existing data on CMR-determined TAPSE measure the maximum apical excursion of the lateral tricuspid annular plane in the four-chamber view with reference to a point in the apex of the right ventricle. Whereas to determine TAPSE with echocardiography, the M-mode curser is oriented from outside the right ventricle to the junction of the tricuspid valve plane with the RV free wall using the apical four-chamber view. Therefore, in our opinion a CMR approach to measure TAPSE with a reference point outside the right ventricle would be more comparable with echocardiography. Moreover, patients with HCM have a hypercontractile right ventricle and a significantly greater apical torsion at end systole [15] compared with other clinical conditions. Thus, use of a reference point in the RV apex might distort the longitudinal TAPSE measurement since the RV apex might leave the central axis plane. Furthermore; no information is so far available for determination of TAPSE in patients with HCM.

Thus, the aim of our study was to evaluate the accuracy of semi-quantitative methods to assess RV function using TAPSE with a reference point within the ventricle (TAPSE_in_) or with one outside the ventricle (TAPSE_out_) and RV fractional shortening (RVFS) derived from CMR images in comparison to RV volumetry in patients with HCM. Furthermore, we performed a subgroup analysis in patients with hypertrophic obstructive cardiomyopathy (HOCM), as well as in patients with hypertrophic non-obstructive cardiomyopathy (HNCM) to test these measurement approaches.

## Methods

### Study population

A total of 105 consecutive patients with HCM referred for CMR were enrolled at our department between February 2003 and August 2012. The population included 60 patients as reported earlier [16]. All patients with HCM were diagnosed based on conventional criteria [17]. Patients with a pressure gradient >30 mmHg at rest or after provocation with either Valsalva manoeuvre and/or after application of nitroglycerine were classified as HOCM.

Twenty age-and sex-matched healthy subjects served as controls and satisfied the following criteria: normal physical examination, normal blood pressure (systolic <130 mmHg and diastolic <85 mmHg), normal ECG findings, no history of chest pain or dyspnoea, no diabetes, no hyperlipidaemia and normal 2D echocardiography and Doppler examination. None of the control subjects were on medication. Exclusion criteria for healthy controls were the presence of signs or symptoms of cardiac diseases, hypertension, diabetes, smoking, or participation in competitive sports.

All patients and volunteers gave informed consent and the study was approved by the local ethics committee.

### CMR image acquisition

All studies were performed using a 1.5 Tesla whole body imaging system (Magnetom Avanto and Sonata, Siemens Healthcare Sector, Erlangen, Germany) using a four-element (Sonata) or a six-element (Avanto), phased-array body coil. Images were acquired with ECG gating and during breath-holds in mild expiration.

Cine images were acquired using a balanced segmented steady state free precession (trueFISP) sequence in three long-axis views (two-, three-, and four-chamber view) and in multiple short-axis views, covering the entire left ventricle from base to apex. Typical image parameters were: TE = 1.2 ms, TR = 3.2 ms, temporal resolution 35 ms, in-plane spatial resolution 1.4 × 1.8 mm2, slice thickness 8 mm, interslice gap 2 mm.

### CMR image analysis

CMR data were analysed using commercially available software (Argus, Siemens Healthcare).

For RV volumetry the short-axis cine loops were reviewed and the end-diastolic and end-systolic frames were identified for each short-axis section position. The first frame of each cine image showing the largest RV cavity area was defined as end-diastole. The phase of end-systole was defined as the frame showing the smallest cavity area. The most basal section was the area of the RV outflow tract below the level of visible pulmonary valve tissue. At the inflow portion of the RV, only the area of the blood pool surrounded by trabeculated ventricular myocardium was included in the RV volume. Identification of the tricuspid valve annulus was facilitated by the simultaneous display of a cross-referenced four-chamber cine image. Endocardial contours were outlined manually at the boundary between the blood pool and the compact myocardium on each end-diastolic and end-systolic short-axis view CMR image. Trabeculations and papillary muscles were included as part of the RV volume. The workstation calculated end-systolic and end-diastolic RV volumes using a modification of the Simpson rule. The RV stroke volume was calculated by subtracting the end-systolic volume (ESV) from the end-diastolic volume (EDV). The RV ejection fraction (RVEF) was calculated by dividing the stroke volume by the EDV.

**Fig. 1:**
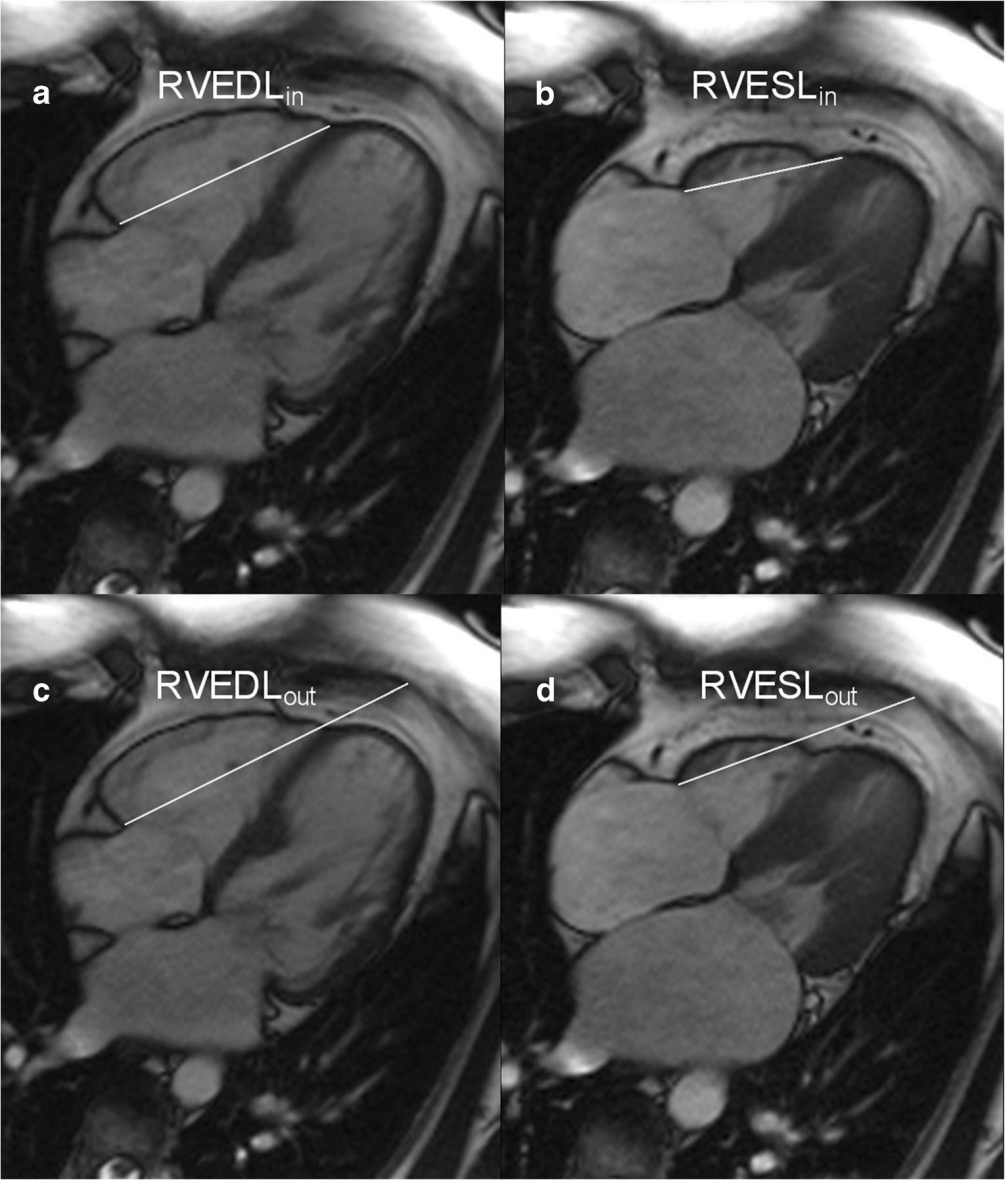
Schematic figure of TAPSE_in_ (Panel **a** and **b**) and TAPSE_out_ (Panel **c** and **d**) measurement using a four-chamber cine image. Two separate reference lines are drawn in diastole (right ventricular end-diastolic length [RVEDL]) and systole (right ventricular end-systolic length [RVESL]) from the basal lateral tricuspid annulus to a reference point in the right ventricular apex: TAPSE_in_ (Panel **a** and **b**) or a reference point outside the right ventricle: TAPSE_out_ (Panel **c** and **d**) *Abbreviations*: *TAPSE* tricuspid annular systolic excursion

On the four-chamber view, the distance between the cutting edge of the tricuspid annulus with the RV free wall and the RV apex or a reference point outside the RV apex were measured in end-diastole (end-diastolic length (EDL)_in_ or EDL_out_) and end-systole (end systolic length (ESL)_in_ or ESL_out_). The point outside the RV apex was chosen in extension to the RV apex and had to stay unchanged at end-diastole and end-systole. In order to ensure that the reference point outside the RV apex stayed unchanged during end-systole, we left the curser at the point of the chosen reference point while scrolling from end-diastole to end-systole. TAPSE_in_ (Fig. 1a, b) was defined as the difference between EDL_in_ and ESL, TAPSE_out_ (Fig. c, d) as the difference between EDL_out_ and ESL. The RVFS was calculated as follows: RVFS (%) = [(EDL_in_ –ESL_in_)/EDL _in_] × 100 [12].

To determine interobserver and intraobserver reliability, TAPSE_in_ and TAPSE_out_ were measured in the first 20 HCM patients twice by the first observer and once by a second observer. A period of at least 1 week passed between the two measurements made by the first observer.

### Statistical analysis

All data are presented as a mean ± standard deviation. Continuous parameters were compared using a two-tailed student’s *t*-test. Pearson’s correlation coefficients (r) were calculated for the relation between TAPSE_in_, TAPSE_out_, RVFS and RVEF. Additionally, Pearson’s r was transformed to z using the following formula: z = 0.5*[ln(1 + r)-ln(1-r)], where ln is the natural logarithm. Fisher’s z was then used for computing 95 % confidence intervals (CI) on the difference between correlations. Lower limit of the 95%CI = z-1.96*σ, upper limit of the 95%CI = z + 1.96*σ, σ = 1/√(n-3), whereas r is the correlation coefficient and n = number of observations. Agreement between the measurement of TAPSE_in_ and TAPSE_out_ was assessed using the Bland-Altman method of analysis [18], providing the mean difference between the measurements (d = bias), the standard deviation (SD) of the differences and the limits of agreement (d ± 1.96SD, where SD = standard deviation of the differences). [18] A one one-sample *t*-test against zero for the statistical significance of the observed differences in intraobserver and interobserver variability was performed.

The ability of TAPSE_in_, TAPSE_out_, and RVFS to predict an RVEF <45 % was calculated using receiver-operating characteristic (ROC) analysis [19]. All results were considered statistically significant when *p* < 0.05. Analyses were performed with Statistical Package for Social Sciences (SPSS for windows 14.0, Chicago, IL, USA).

## Results

Baseline patients’ and RV CMR characteristics are displayed in Table 1. In the whole cohort of patients with HCM, the mean RVEF was normal with a mean of 63 ± 10 %. Only 6 (5.7 %) patients presented with an impaired RVEF <45 %; 4 (3.8 %) of them had an impaired RVEF < 40 %. None of the patients had an RVEF <35 %. Right ventricular end-systolic volume index (RV-ESVI) was higher and RVEF was lower in patients with HNCM compared with those with HOCM. In the whole cohort of patients with HCM (*n* = 105), RVEF determined by CMR revealed a weak correlation with TAPSE_in_ (*r* = 0.31, 95 % CI 0.13–0.51, *p* = 0.001) and RVFS (*r* = 0.35, 95 % CI 0.17–0.56, *p* = 0.0002) but a moderate correlation with TAPSE_out_ (*r* = 0.57, 95 % CI 0.46–0.84, *p* < 0.0001). Comparison of TAPSE_in_ and TAPSE_out_ showed a weak correlation (*r* = 0.35, *p* = 0.0002).

**Table 1:** Patient characteristics and right ventricular CMR parameter

	Healthy controls *n* = 20	HCM *N* = 105	*p*-value Controls vs HCM	HOCM *n* = 40	HNCM *n* = 65	*p*-value HOCM vs HNCM
Age (years)	53 ± 13	56 ± 15	0.4	58 ± 13	54 ± 16	0.2
Male gender (n,%)	12 (60)	63 (60)	0,99	25 (63)	38 (58)	0.9
Height (m)	1.72 ± 0.8	1.73 ± 0.9	0.7	1.74 ± 0.9	1.72 ± 0.9	0.1
Weight (kg)	78 ± 18	83 ± 17	0.3	86 ± 14	81 ± 18	0.1
LVOT gradient	–	40 (38)	–	40 (100)	–	–
• At rest	–	20 (19)	–	20 (50)	–	–
• After provocation	–	20 (19)	–	20 (50)	–	–
RV-EF (%)	58 ± 4	63 ± 10	0.05	66 ± 9	61 ± 11	0.01
TAPSE_in_ (cm)	2.0 ± 0.3	2.5 ± 0.9	0.01	2.7 ± 0.8	2.4 ± 1.0	0.1
TAPSE_out_ (cm)	1.9 ± 0.2	1.8 ± 0.5	0.1	1.8 ± 0.5	1.8 ± 0.5	0.8
RVFS (%)	24 ± 3	32 ± 10	0.0002	34 ± 10	31 ± 10	0.1
RV-EDVI (ml/m^2^)	68 ± 10	69 ± 22	0.9	66 ± 17	70 ± 24	0.3
RV-ESVI (ml/m^2^)	28 ± 6	27 ± 12	0.4	23 ± 9	28 ± 13	0.04
RV-SVI (ml/m^2^)	40 ± 6	42 ± 12	0.6	44 ± 10	41 ± 13	0.2

*Abbreviations*: *EDVI* end-diastolic volume index, *EF* ejection fraction, *ESVI* end-systolic volume index, *FS* fractional shortening, *HCM* hypertrophic cardiomyopathy, *HNCM* hypertrophic non-obstructive cardiomyopathy, *HOCM* hypertrophic obstructive cardiomyopathy, *LVOT* left ventricular outflow tract, *RV* right ventricular, *TAPSE*_*in*_ tricuspid annular plane systolic excursion with a reference point within the right ventricle, *TAPSE*_*out*_ tricuspid annular plane systolic excursion with a reference point outside the right ventricle

In the subgroup analysis of patients with HOCM (*n* = 40), RVEF showed a significant but weak correlation with TAPSE_out_ (*r* = 0.36, 95 % CI 0.14–0.62, *p* = 0.02) and no correlation with TAPSE_in_ (*r* = 0.05, 95 % CI −0.19–0.29, *p* = 0.7) and RVFS (*r* = 0.20, 95 % CI −0.04–0.44, *p* = 0.2). In patients with HNCM (*n* = 65), there was a moderate correlation between RVEF and TAPSE_out_ (*r* = 0.57, 95 % CI 0.49–0.81 *p* < 0.0001) but a weak correlation with TAPSE_in_ (*r* = 0.39, 95 % CI 0.25–0.57, *p* = 0.001) and RVFS (*r* = 0.38, 95 % CI 0.24–0.56, *p* = 0.002). In healthy controls, there was a good correlation between RVEF and both TAPSE_in_ (*r* = 0.69, 95 % CI 0.61–1.09, *p* = 0.001) and RVFS (*r* = 0.55, 95 % CI 0.38–0.86, *p* = 0.01). Furthermore there was a strong correlation between RVEF and TAPSE_out_ (*r* = 0.80, 95 % CI 0.86–1.34, *p* < 0.0001).

**Fig. 2:**
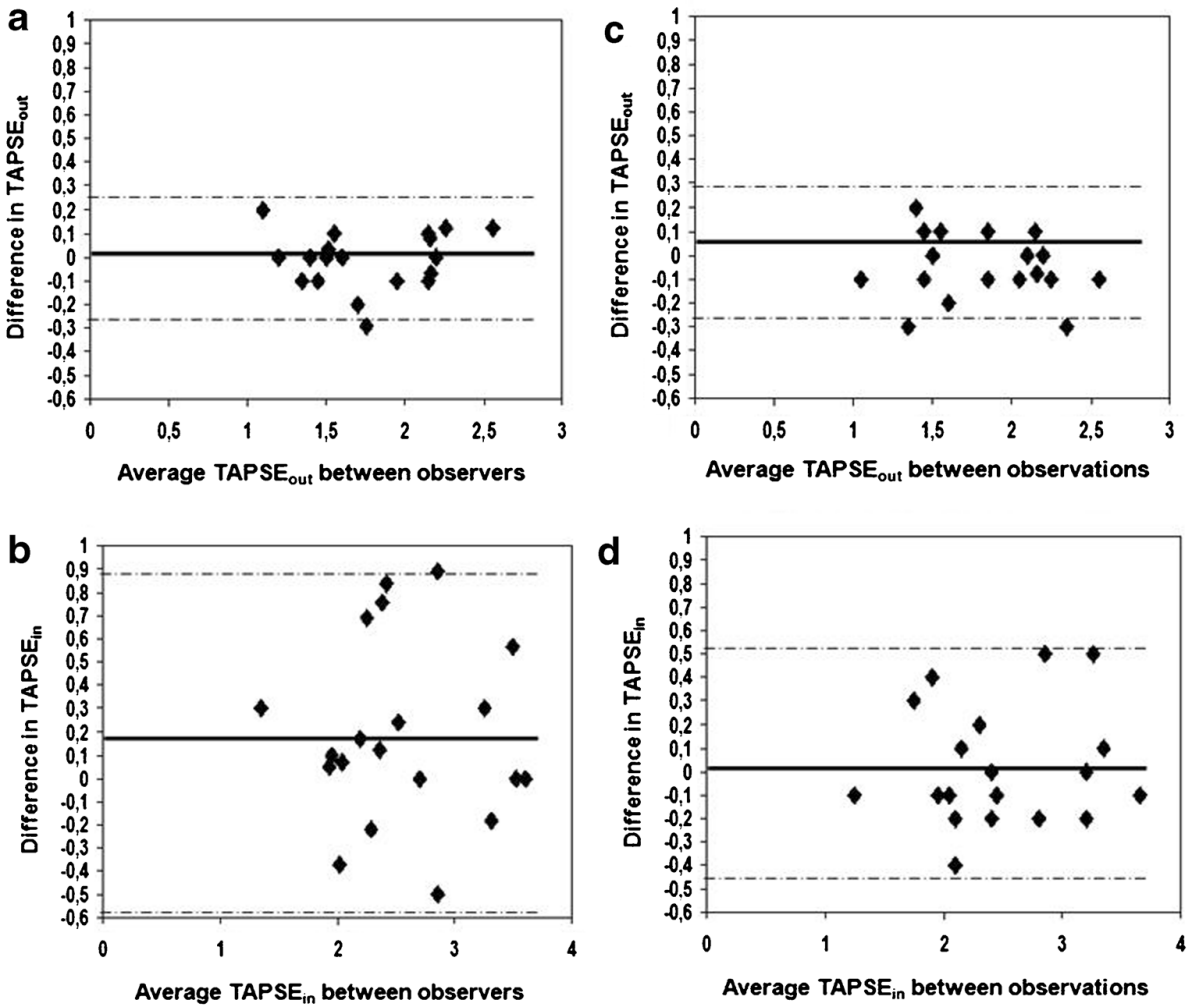
Bland-Altman plots depict interobserver and intraobserver agreement regarding TAPSE_out_ (**a**, **c**) and TAPSE_in_ (**b**, **d**). On each plot, the solid line represents mean value of the differences between measurements between two observers (**a**, **b**) or between two observations (**c**, **d**). Dotted lines represent ± 2 SD. The mean value of the two measurements is plotted along the x-axis and the difference between two observer or observations is plotted along the y axis *Abbreviations*: *SD* standard deviation, *TAPSE* tricuspid annular systolic excursion

The agreement between TAPSE_out_ and TAPSE_in_ was analysed (Table 2). The limits of interobserver as well as intraobserver agreement for TAPSE_out_ measurements were narrower than those for the TAPSE_in_ measurements (Table 2, Fig. 2). Additionally, the coefficients of repeatability were lower for the TAPSE_out_ measurements, indicating better reproducibility (Table 2).

**Table 2:** Interobserver and intraobserver reproducibility of TAPSE and TAPSE in 20 patients with hypertrophic cardiomyopathy

	TAPSE_out_			TAPSE_in_			
	Mean ± SD	Limits of agreement	Coefficient of repeatability	Mean ± SD	Limits of agreement	Coefficient of repeatability	*p*-value
Interobserver variability	−0.01 ± 0.12	−0.24 to +0.22	0.23	0.19 ± 0.38	−0.56 to +0.94	0.77	0.001
Intraobserver variability	−0.05 ± 0.13	−0.31 to +0.26	0.26	0.02 ± 0.25	−0.48 to +0.51	0.5	0.04

*Abbreviations*: *SD* standard deviation, *TAPSE* tricuspid annular systolic excursion

**Fig. 3:**
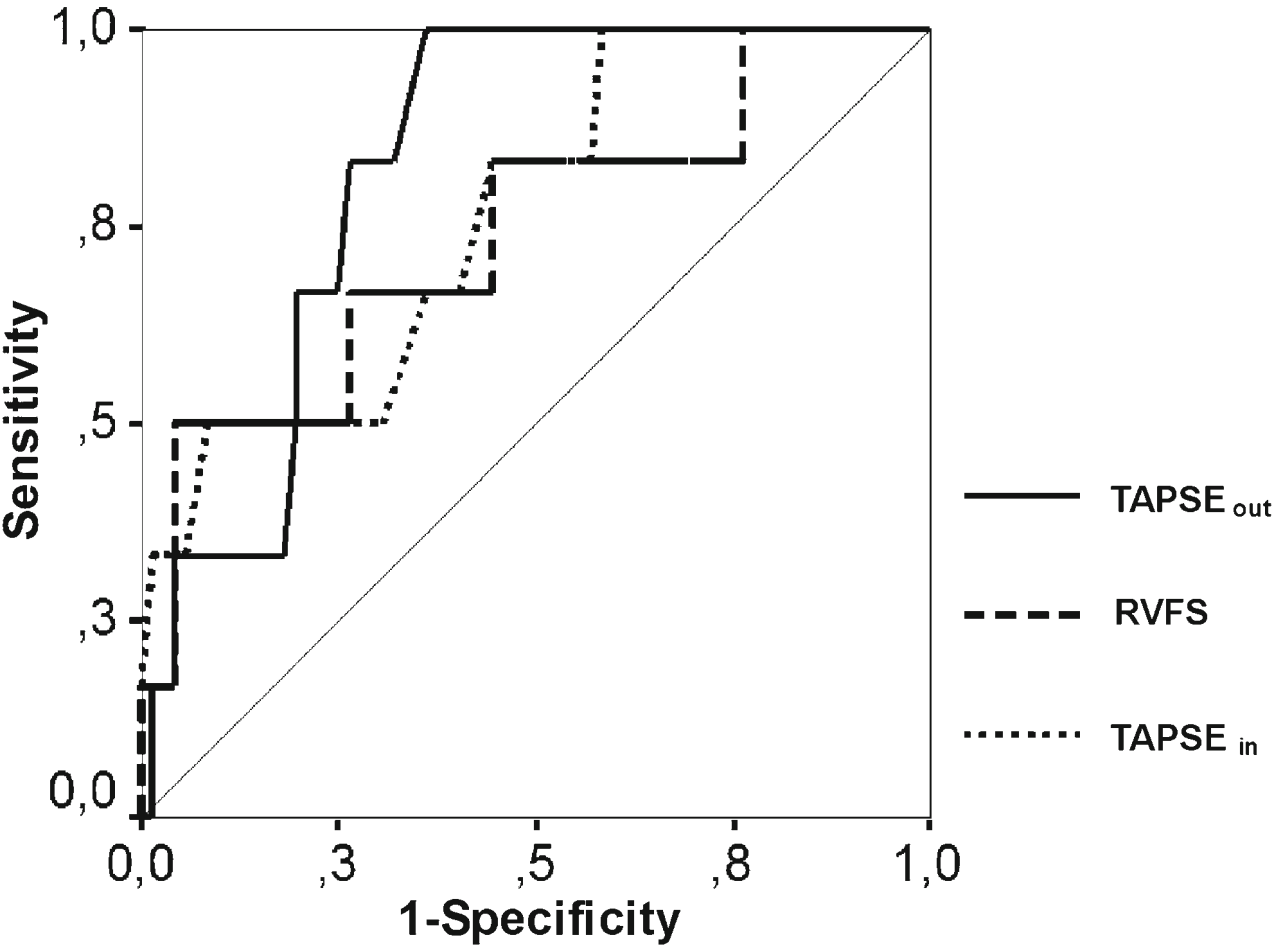
Receiver-operating characteristic curve of TAPSE_out_ (*solid line*), TAPSE_in_ (*dotted line*) and RVFS (*dashed line*) to indicate right ventricular ejection fraction of less than 45 %. Area under the curve was 0.83 for TAPSE_out_,0.77 for TAPSE_in_ and 0.74 for RVFS. *Abbreviations*: *SD* standard deviation

Figure 3 shows the ROC curve analysis of TAPSE_out_, TAPSE_in_ and RVFS in the whole cohort of patients with HCM. The ability to predict an RVEF < 45 % was best for TAPSE_out_ (AUC = 0.83, *p* = 0.01). The area under the ROC curve for TAPSE_in_ (AUC 0.77, *p* = 0.03) and RVFS (AUC 0.74, *p* = 0.05) were inferior. With a cut-off value TAPSE_out_ of 1.5 cm, the sensitivity and specificity to detect an RVEF ≤ 45%was 67 % and 82 %, respectively.

## Discussion

Our study demonstrates that CMR-derived TAPSE measurements using a reference point in the RV apex (TAPSE_in_) are not suitable in patients with HCM. CMR TAPSE measurements with a reference point outside the right ventricle (TAPSE_out_) showed a good correlation with the standard RV volumetric approach in patients with HNCM, but only a weak correlation in patients with HOCM. Comparison of TAPSE_in_ and TAPSE_out_ showed a weak correlation in the whole population.

In patients with HCM, RV wall thickening is common and it is well known that the hypertrophic process in HCM is diffuse [2–5]. Therefore, detection of RV dysfunction might also be important in these patients. A study by McKenna et al. [2] in patients with HCM reported not only a significant association between RV involvement and severity of symptoms but they also found an increased incidence of ventricular tachycardia and supraventricular arrhythmias in patients with HCM and concomitant RV involvement. Moreover, patients with HCM and RV hypertrophy were assumed to present more often with atrial fibrillation [20]. Even in the absence of atrial fibrillation, an increased risk of pulmonary embolism due to RV aneurysm formation associated with a poorer prognosis has been shown [21]. These findings highlight the importance to detect RV involvement in patients with HCM.

Echocardiographically assessed TAPSE represents a quick semi-quantitative approach to assess information about the RV function [22–24]. Using echocardiography, the two-dimensional M-mode curser is positioned on the lateral tricuspid annulus near the free RV wall in the apical four-chamber view and aligned as close as possible to the apex of the heart. Recent studies using CMR introduced a modified TAPSE approach with a reference point in the RV apex (TAPSE_in_). This approach showed a good correlation with quantitative assessment of RV function in patients with heart failure due to ischaemic heart disease [12, 14, 25], pulmonary artery hypertension [12, 13], Brugada syndrome [12], dilated cardiomyopathy [25] and valvular or congenital heart disease [25]. However, in patients with surgically repaired tetralogy of Fallot CMR-derived TAPSE measurement with a reference point in the RV apex was not a reliable measure of RVEF [26]. The authors hypothesised that the poor correlation between TAPSE and RVEF assessed by CMR in repaired tetralogy of Fallot patients may be due to the increased function in the apical RV slices and an altered contraction pattern in these patients.

The two approaches to determine TAPSE using either echocardiography or CMR-derived TAPSE with a reference point in the RV apex (TAPSE_in_) are different. The echocardiographic TAPSE measurement takes into account only movements of the tricuspid annular base. The CMR approach uses a reference point in the RV apex. Thus, the longitudinal TAPSE measurement may be falsified by the movement of the RV apex out of the central plane. Therefore, we introduced a new CMR method to determine TAPSE with a reference point chosen outside the RV which is unaffected by the contraction and the torsion of the apex and is more comparable with the echocardiographic measurements. In this context our results showed that in patients with HCM, TAPSE measurements with a fixed reference point outside the RV (TAPSE_out_) correlated best with the standard RV volumetric approach and seem to reflect more accurately the tricuspid annular systolic excursion than the measurements with the reference point within the right ventricular apex (TAPSE_in_). These findings were more pronounced in the subgroup analysis. In patients with HNCM there was a good correlation between RVEF and TAPSE_out_ and a weak correlation with TAPSE_in_ and RVFS. Furthermore, in patients with HOCM, RVEF showed a significant but weak correlation with TAPSE_out_ and no significant correlation with TAPSE_in_ and RVFS. One could assume that in patients with HCM and especially in patients with HOCM who have a predominantly hypercontractile right ventricle and a significantly greater apical torsion at end-systole [15], the impact of the apical torsion on TAPSE measurements is not negligible.

Similarly to the results of Nijveldt et al. [12] our healthy subjects also showed a good correlation to the three-dimensional volumetric approach using TAPSE_in_. However, even in our healthy population, CMR-derived TAPSE_out_ measurements revealed a better correlation to RVEF than the TAPSE_in_ or RVFS indicating that inclusion of the RV apex as a reference point may also affect TAPSE measurements in healthy volunteers. Furthermore, Nijveldt et al.[12] validated TAPSE and RVFS as a screening tool to identify patients with RV dysfunction in several clinical conditions. These measures showed good performance, but also had limitations. For routine RV screening TAPSE and RVFS seemed to be easy and reliable methods to identify these patients but they were not sufficient to detect subtle changes in RV function. Comparable with these findings, our results also showed a good performance of all semi-quantitative methods, best for TAPSE_out_, to detect RV dysfunction. However, they were also not suitable for the detection of small changes in RV function.

To the best of our knowledge the present study is the first to compare CMR-derived TAPSE measurements using either a reference point inside or outside the RV apex. To date, no data were available on which method to use to achieve the most reliable and reproducible results for RV longitudinal function analysis using CMR. The clinical value of a specific analysis method is determined not only on the basis of its accuracy but also on its reproducibility. Therefore, we sought to analyse which method was superior in terms of reproducibility. The interobserver and intraobserver variability of TAPSE_out_ were superior to TAPSE_in_. There are two main reasons for this finding: a) the above-described pronounced apical torsion of the right ventricle in patients with HCM and b) the fact that the right ventricle is also hypertrophied in patients with HCM which makes it more difficult to identify the RV apex, particularly in end-systole. Furthermore, we investigated for the first time whether a semi-quantitative approach using TAPSE or RVFS derived by CMR is suitable to describe RV function in patients with HCM.

### Clinical implications

In terms of acquisition, images required for the gold standard volumetric 3D approach and for the semi-quantitative 2D analysis are actually both part of the routine CMR imaging protocol. The difference is mainly due to the post-processing. The 3D approach requires outlining the endocardial border of the right ventricle on each slice of the short-axis stack which takes up to 10 min, depending on the experience of the observer, whereas the 2D approach only requires to determine in end-diastole and end-systole the distance between the cutting edge of the tricuspid annulus with the RV free wall and a reference point outside the right ventricle which can be done in 1 min. Thus, it is advantageous to have a quick 2D screening tool to identify patients with RV dysfunction and to select patients in whom a more detailed analysis would be beneficial. Furthermore, 2D TAPSE or RVFS measurements can easily be assessed by less experienced readers whereas 3D RV volumetry requires more experience.

### Limitations

In our cohort of patients with HCM, there was only a small number of patients with reduced RVEF. Thus, the suggested TAPSE cut-off values to discriminate between patients with normal and reduced RVEF should be tested in further larger HCM populations.

## Conclusion

CMR-derived TAPSE measurements using a reference point in the RV apex (TAPSE_in_) are not suitable in patients with HCM. CMR TAPSE measurements with a reference point outside the RV (TAPSE_out_) showed a good correlation with the 3D volumetric determined RVEF in patients with HNCM but only a weak correlation in patients with HOCM. TAPSE_out_ might be used to screen for RV dysfunction in patients with HCM. However, the detection of subtle changes in RV function requires the more time-consuming standard 3D quantitative approach.

## References

[1] Guclu A, Germans T, Witjas-Paalberends ER (2013). ENerGetIcs in hypertrophic cardiomyopathy: translation between MRI, PET and cardiac myofilament function (ENGINE study). Neth Heart J.

[2] McKenna WJ, Kleinebenne A, Nihoyannopoulos P, Foale R (1988). Echocardiographic measurement of right ventricular wall thickness in hypertrophic cardiomyopathy: relation to clinical and prognostic features. J Am Coll Cardiol.

[3] Suzuki J, Sakamoto T, Takenaka K (1988). Assessment of the thickness of the right ventricular free wall by magnetic resonance imaging in patients with hypertrophic cardiomyopathy. Br Heart J.

[4] Keeling AN, Carr JC, Choudhury L (2010). Right ventricular hypertrophy and scarring in mutation positive hypertrophic cardiomyopathy. Eur Heart J.

[5] Maron MS, Hauser TH, Dubrow E (2007). Right ventricular involvement in hypertrophic cardiomyopathy. Am J Cardiol.

[6] Morner S, Lindqvist P, Waldenstrom A, Kazzam E (2008). Right ventricular dysfunction in hypertrophic cardiomyopathy as evidenced by the myocardial performance index. Int J Cardiol.

[7] Zemanek D, Tomasov P, Prichystalova P, Linhartova K, Veselka J (2010). Evaluation of the right ventricular function in hypertrophic obstructive cardiomyopathy: a strain and tissue Doppler study. Physiol Res.

[8] Beygui F, Furber A, Delepine S (2004). Routine breath-hold gradient echo MRI-derived right ventricular mass, volumes and function: accuracy, reproducibility and coherence study. Int J Cardiovasc Imaging.

[9] Grothues F, Moon JC, Bellenger NG, Smith GS, Klein HU, Pennell DJ (2004). Interstudy reproducibility of right ventricular volumes, function, and mass with cardiovascular magnetic resonance. Am Heart J.

[10] Grothoff M, Hoffmann J, Lehmkuhl L (2011). Time course of right ventricular functional parameters after surgical correction of tetralogy of Fallot determined by cardiac magnetic resonance. Clin Res Cardiol.

[11] Caudron J, Fares J, Vivier PH, Lefebvre V, Petitjean C, Dacher JN (2011). Diagnostic accuracy and variability of three semi-quantitative methods for assessing right ventricular systolic function from cardiac MRI in patients with acquired heart disease. Eur Radiol.

[12] Nijveldt R, Germans T, McCann GP, Beek AM, van Rossum AC (2008). Semi-quantitative assessment of right ventricular function in comparison to a 3D volumetric approach: a cardiovascular magnetic resonance study. Eur Radiol.

[13] Kind T, Mauritz GJ, Marcus JT, van de Veerdonk M, Westerhof N, Vonk-Noordegraaf A (2010). Right ventricular ejection fraction is better reflected by transverse rather than longitudinal wall motion in pulmonary hypertension. J Cardiovasc Magn Reson.

[14] Speiser U, Hirschberger M, Pilz G (2012). Tricuspid annular plane systolic excursion assessed using MRI for semi-quantification of right ventricular ejection fraction. Br J Radiol.

[15] Young AA, Kramer CM, Ferrari VA, Axel L, Reichek N (1994). Three-dimensional left ventricular deformation in hypertrophic cardiomyopathy. Circulation.

[16] Papavassiliu T, Germans T, Fluchter S (2009). CMR findings in patients with hypertrophic cardiomyopathy and atrial fibrillation. J Cardiovasc Magn Reson.

[17] Maron BJ, Towbin JA, Thiene G (2006). Contemporary definitions and classification of the cardiomyopathies: an American Heart Association Scientific Statement from the Council on Clinical Cardiology, Heart Failure and Transplantation Committee; Quality of Care and Outcomes Research and Functional Genomics and Translational Biology Interdisciplinary Working Groups; and Council on Epidemiology and Prevention. Circulation.

[18] Bland JM, Altman DG (1986). Statistical methods for assessing agreement between two methods of clinical measurement. Lancet.

[19] Hanley JA, McNeil BJ (1983). A method of comparing the areas under receiver operating characteristic curves derived from the same cases. Radiology.

[20] Albanesi Filho FM, Castier MB, Lopes AS, Ginefra P (1997). Is the apical hypertrophic cardiomyopathy seen in one population in Rio de Janeiro city similar to that found in the East?. Arq Bras Cardiol.

[21] Frustaci A, Chimenti C, Natale L (1998). Right ventricular aneurysm associated with advanced hypertrophic cardiomyopathy. Chest.

[22] Kaul S, Tei C, Hopkins JM, Shah PM (1984). Assessment of right ventricular function using two-dimensional echocardiography. Am Heart J.

[23] Koestenberger M, Nagel B, Ravekes W (2011). Tricuspid annular plane systolic excursion and right ventricular ejection fraction in pediatric and adolescent patients with tetralogy of Fallot, patients with atrial septal defect, and age-matched normal subjects. Clin Res Cardiol.

[24] Lemmer J, Heise G, Rentzsch A (2011). Right ventricular function in grown-up patients after correction of congenital right heart disease. Clin Res Cardiol.

[25] Alpendurada F, Guha K, Sharma R (2011). Right ventricular dysfunction is a predictor of non-response and clinical outcome following cardiac resynchronization therapy. J Cardiovasc Magn Reson.

[26] Morcos P, Vick GW, Sahn DJ, Jerosch-Herold M, Shurman A, Sheehan FH (2009). Correlation of right ventricular ejection fraction and tricuspid annular plane systolic excursion in tetralogy of Fallot by magnetic resonance imaging. Int J Cardiovasc Imaging.

